# Newly formed and ruptured infectious intracranial aneurysm in few days following first intracranial aneurysm embolization concurrent with middle cerebral artery occlusion: A regrettable case in a baby child

**DOI:** 10.1002/pdi3.80

**Published:** 2024-06-04

**Authors:** Yunying Yang, Hongtu Ma, Hui Hu, Lusheng Li, Jun Tang

**Affiliations:** ^1^ Department of Neurosurgery Children's Hospital of Chongqing Medical University Chongqing China; ^2^ National Research Center for Child Health and Disorders Chongqing China; ^3^ Chongqing Key Laboratory of Translational Medical Research in Cognitive Development and Learning and Memory Disorders Chongqing China

**Keywords:** infectious intracranial aneurysm, infective endocarditis, intracerebral hemorrhage, pediatric aneurysms

## Abstract

Infectious intracranial aneurysm (IIA) and embolic cerebral infarction are well‐known devastating complications of children suffering infective endocarditis. In this report, we describe a successfully embolized IIA concurrent with bilateral middle cerebral artery (MCA) occlusion. Unfortunately, a newly formed IIA located in the contralateral MCA bifurcation ruptured at the seventh day following embolization. A 6‐month‐old female child was admitted to hospital 3 days following acute right limb mobility disorder. An interventional surgery history of congenital heart disease was confirmed. She was immediately started on antibiotic therapy and the computed tomography agiography (CTA) scan showed occlusion of the upper branch of the left MCA. Unfortunately an IIA was located in the distal artery region (DAR) of the ipsilateral anterior cerebral artery. Angiography (digital subtraction angiography) was performed and the DAR IIA was embolized by OnyX‐18 with Magic 1.2 Fr. microcatheter. On the sixth day, magnetic resonance imaging during the hospital stay showed reduced infarction area with no other special sign. Desperately, a major seizure with opisthotonos attacked the baby on the seventh day after embolization. An immediate CTA scan showed massive hematoma in the right basal ganglia and a ruptured bifurcate aneurysm of the right MCA. The parents refused positive treatment and discharged in considering the critical situation. It should be noted that IIA can be fast formed anywhere in cerebral artery and dynamic angio‐image should be performed as supervision.

## 1 INTRODUCTION

Since the first description of infectious intracranial aneurysm (IIA) was presented by Church in 1869, embolic cerebral infarction and IIA have been recognized as common complications of infective endocarditis (IE) in children.^1^, ^2^ Actually, acute ischemia stroke (AIS) attacks frequently than IIA in children with IE. The International Pediatric Stroke Study identified 30% AIS of children with cardiac disorders, among which 25% AIS attacked in the perioperative period.^3^ IIA concurrent with AIS secondary to IE has also been reported.^4^ IIAs are pseudoaneurysm and relatively rare lesions but associated with high mortality when they rupture. From epidemiology, IIA presents following acute inflammatory reaction of the adventitia that spreads into the muscular layer, resulting in disruption of both internal elastic membrane and intima.^5^ In general, IIAs account for approximately 15% of all pediatric intracranial aneurysms and are located within the anterior circulation (75%–93%).^6^, ^7^

Most importantly, it is very difficult to supervise the forming process of IIA and assess the state before rupture within a short period of time. Here, we reported a regrettable baby child case of newly formed and ruptured IIA in few days following first intracranial aneurysm embolization concurrent with middle cerebral artery (MCA) occlusion. To our knowledge, it is the first presentation of the newly formed IIA that ruptured within a short time and proved by digital subtraction angiography (DSA).

## 2 CASE REPORT

**FIGURE 1:**
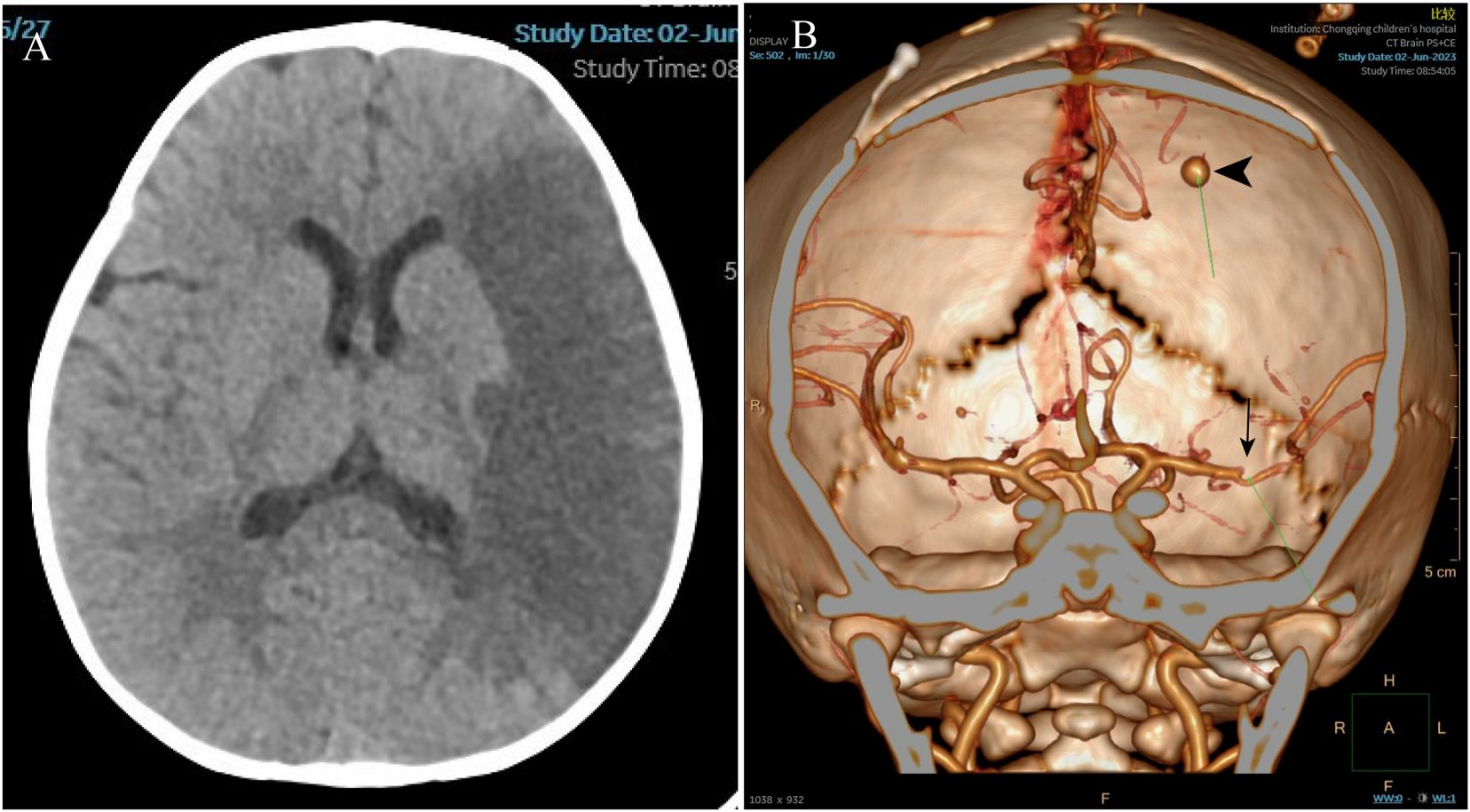
In‐department imaging examination at admission: (A) CT scan presented embolic cerebral infarction of left temporal lobe. (B) CTA scan presented upper branch occlusion of middle cerebral artery (MCA) (black arrow) and infectious intracranial aneurysms (IIA) located in distal artery region (DAR) of ipsilateral MCA (black arrow head). CTA, computed tomography angiography.

**FIGURE 2:**
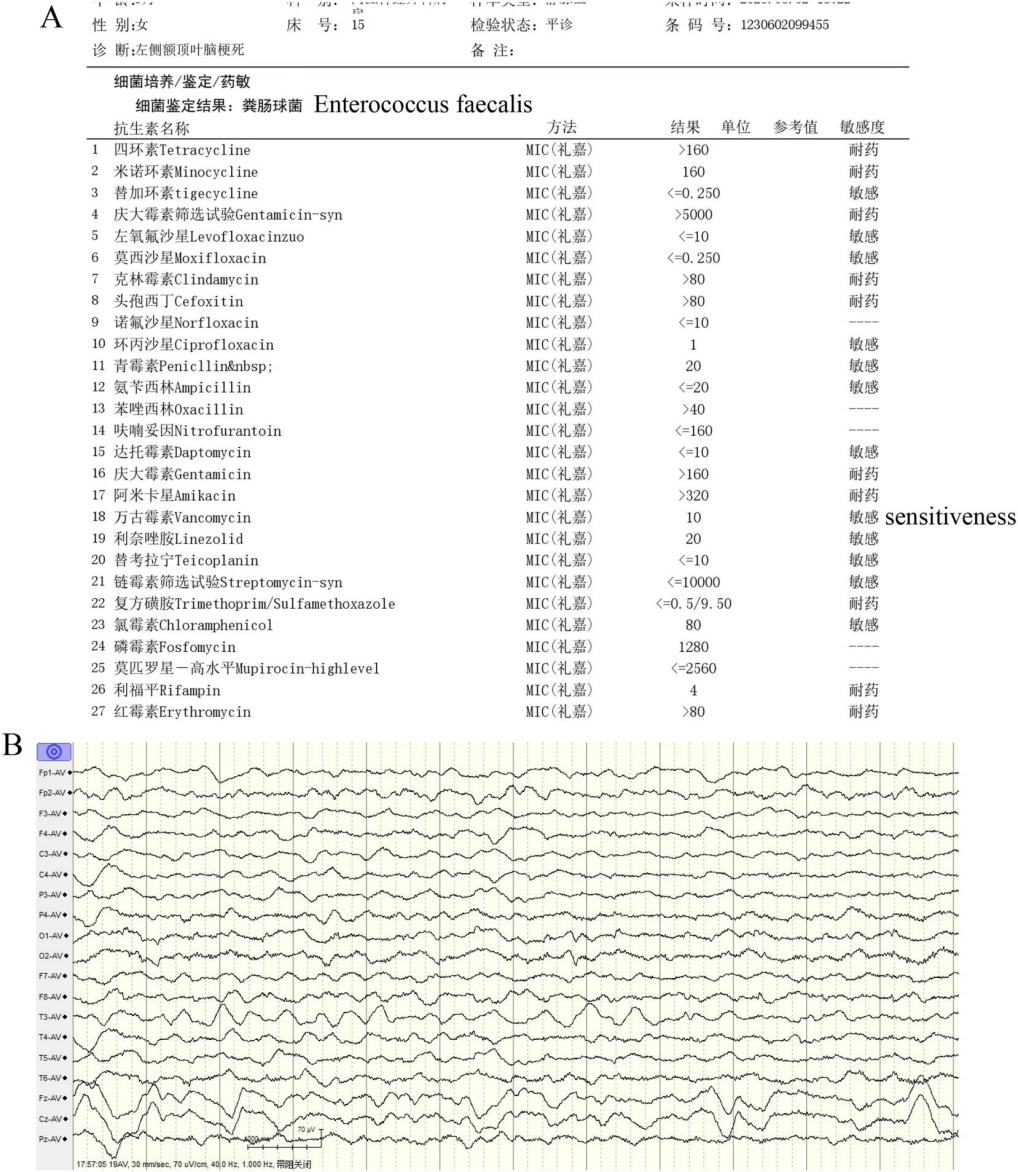
(A) Blood culture detected with bacterial identification result: *enterococcus faecalis* positive and vancomycin sensitive when admitting. (B) electroencephalogram (EEG) detected no epileptic wave during seizure attack.

A 6‐month‐old female child with surgery history of congenital heart disease, 2 months ago had a 3‐day history of right limb mobility disorder. No seizure was presented and the electroencephalogram (EEG) was negative. In‐department CT/computed tomography angiography (CTA) scan presented embolic cerebral infarction with upper branch occlusion of the left MCA, unfortunately an IIA was located in the distal artery region (DAR) of ipsilateral MCA (Figure 1A,B). Meropenem and vancomycin were immediately injected intravenously for antibiosis and blood culture detected *enterococcus faecalis* (Figure 2A). Since the AIS happened 3 days before hospital, there was no sign for alteplase or mechanical thrombectomy. While the IIA in DAR should be positively treated in case of rupture, we arranged a selective operation of intervascular embolization of IIA. Unfortunately, the baby suffered multiple seizure with negative EEG (Figure 2B) the day before operation but it was controlled by medication. The operation was postponed 3 days later when no seizure occurred.

**FIGURE 3:**
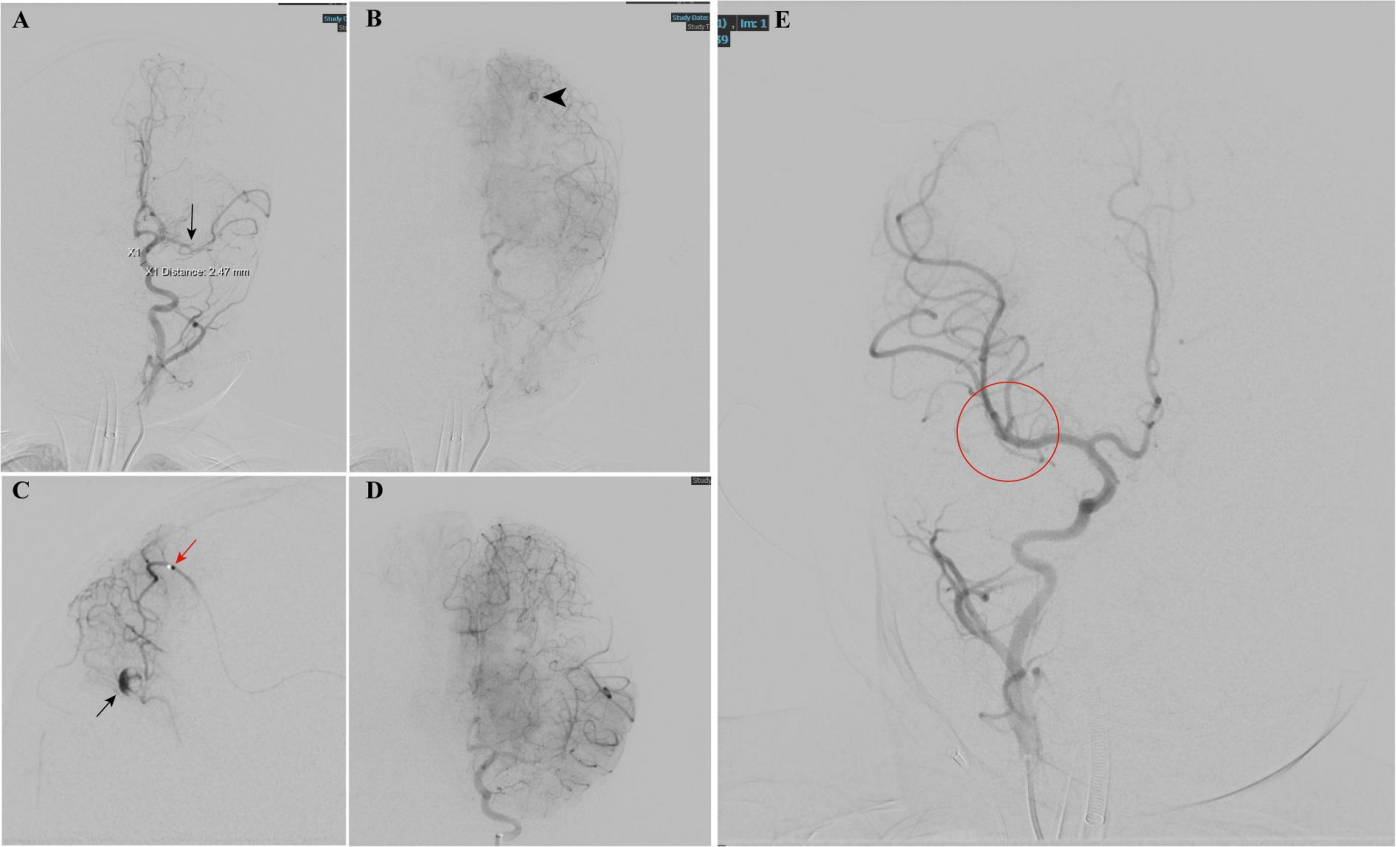
(A) digital subtraction angiography (DSA) presented upper branch occlusion of middle cerebral artery (MCA) (black arrow). (B) DSA presented Infectious intracranial aneurysms (IIA) located in distal artery region (DAR) of the anterior artery in the late arterial phase. (C) Selective angiography performed by navigated Magic 1.2 Fr. microcatheter (red arrow) and the IIA developed (black arrow). (D) The IIA is invisible after embolized by OnyX‐18. (E) DSA in right ICA presented no abnormal image of vessels, especially bifurcation of MCA (red circle). ICA, internal carotid artery.

During intravascular treatment, an IIA located in DAR of anterior artery concurrent with occlusion of the upper branch of left MCA was captured (Figure 3A,B) by DSA. No artery occlusion and aneurysms were found in other arteries (Figure 3C). Trans‐arterial embolization was performed in condition of Siemens Axiom Artis system (Siemens AG, Medical Solutions Erlangen, Germany) under general anesthesia. A 4‐Fr. envoy guiding catheter (Cordis, Miami, FL, USA) was inserted into the left internal carotid artery via the right trans‐femoral approach. A 1.2‐Fr. magic microcatheter (Balt, Montmorency, France) was navigated to the parent artery of IIA as far as possible under roadmap (Figure 3D). A selective angiography by 1 ml syringe proved that the IIA located far distal segment of left anterior artery and the tip of microcatheter cannot navigate anymore; the IIA was embolized by OnyX‐18 (ev3, Irvine, CA, USA). The immediate DSA demonstrated no residual of aneurysm and the occlusion of left MCA stayed still (Figure 3E).

**FIGURE 4:**
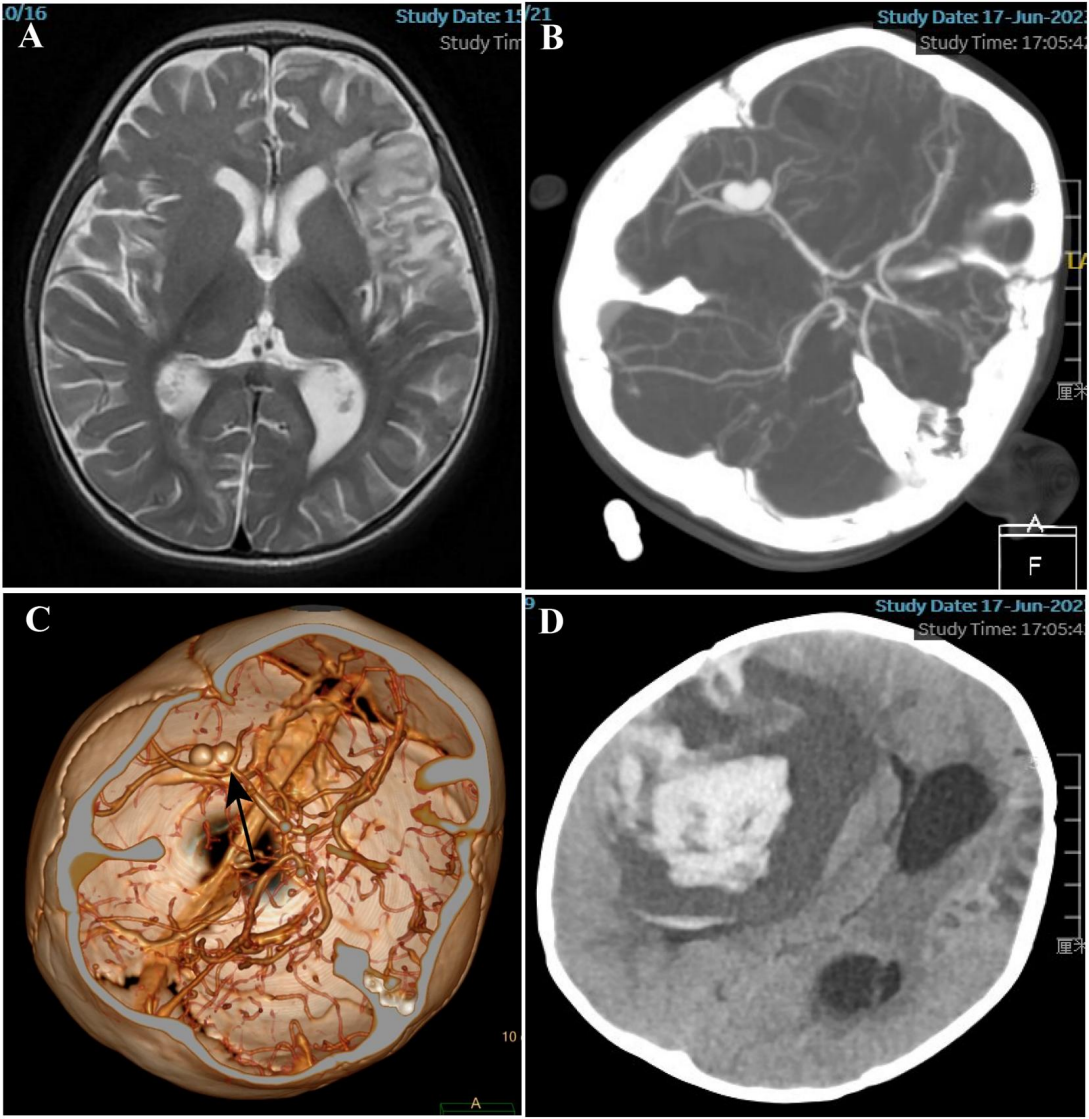
(A) MRI image 6days after embolization. (B, C) CTA scan immediately following the seizure attack 7days after embolization and the newly formed Infectious intracranial aneurysms (IIA) presented (black arrow). CT scan presented a massive hematoma located in the right basal ganglia. CTA, computed tomography angiography; MRI, magnetic resonance imaging.

After intervascular operation, the baby was treated with anti‐seizure and antibacterial medication sequentially. Twice blood culture presented negative after the first positive one when arriving at the hospital. No seizure occurred and the disorder of right limb mobility recovered at the sixth day following operation. Magnetic resonance showed that the infarction area of left temporal lobe decreased and no other abnormal signal was detected in any other lobes (Figure 4A). Unfortunately, the baby suffered a sudden major seizure with opisthotonos in the morning of the seventh day after operation. The patient's condition changed fast with low oxygen saturation and tracheal intubation was performed. Immediately following emergent operation, a CTA scan showed that a big aneurysm approximately 11.6 mm * 8.6 mm located in the middle branch artery of the right MCA bifurcation (Figure 4B,C). CT scan presented a massive hematoma in the right temporal lobe with serious edema and obvious midline shift (Figure 4D). After all, the parents decided to abandon further treatment in considering surgical risk, terrible prognosis, and possible another new‐formed IIA in the future. The baby was taken home and died the next day. We confirm that informed consent was obtained from the patients' parent to report this case in public.

## 3 DISCUSSION

To our knowledge, it is the first presentation of the newly formed IIA located far different from the first one and ruptured within a short time proved by DSA. The present case demonstrated three important clinical issues. First, bacterial thrombus can induce IIA and AIS simultaneously. Second, IIA associated with IE may newly‐form anytime in the early period and rupture within days. Third, vascular condition should be dynamically supervised since one IIA was presented. Although headaches and non‐hemorrhagic deficits were the most common presenting complaints in infectious aneurysms,^8^ The rupture incidence of IIA reported was 13%–17%.^5^ Considering the tremendous prognosis of aneurysmal rupture, we recommend positive operation of IIA in the early stage.

Three types of IIA were classified according to their pathophysiology.^9^ The first type was intravascular origin formed as a result of embolization from bacterial endocarditis, like the present case. The second one occurs by extension of a neighboring infection or vessel wall invasion from the contiguous infected anatomical structure and centripetal migration toward the elastic intimal layer, such as parasite‐induced IIA. The third type is the so called primary or cryptogenic mycotic aneurysm that no obvious inflammatory lesion can be found. *Staphylococcus* and *streptococcus* species are the most common causative organisms in IIAs; among them, *staphylococcus aureus* is the most commonly cultured pathogen.^10^ *Streptococcus* viridans and *staphylococcus aureus* are reported to be responsible for 57%–91% of IIAs.^5^ In the present case, the first blood culture presented *enterococcus faecalis* and the result became negative at the last twice culture because of antibacterial medication. Infective endocarditis is the most serious complication following transcatheter aortic valve replacement (TAVR), even the procedures and equipments are more advanced.^11^ Mycotic aneurysms were recorded in 5% of IE, which is associated with the migration of the pathogen to vasa vasorum or intraluminal space.^12^ Considering the poor prognosis of IE following TAVR, prevention should be the cornerstones for patients' life. Although most recommendations advocate periprocedural antibiotic prophylaxis in patients undergoing TAVR, uncertainties remain about the most appropriate medications for infection control. On the other hand, the greater exposure to health care interventions is reported to result in high risk of IE after TAVR. Some investigators have suggested the importance of limiting health care associated procedures.^11^

The natural history of untreated IIA is tremendously detrimental with high incidence rate of rupture. Ducruet et al. reported that the incidence rate of aneurysm rupture is 72% in patients with IIAs and the rupture‐induced hematoma or subarachnoid hemorrhage may be the first clinical manifestation of illness.^10^, ^13^ The high rate of IIA rupture resulted from the morphological characteristics of pseudoaneurysms with thin wall and no obvious neck. The inflammatory reaction of adventitia that spreads into the vascular muscular layer leads the vascular wall friable, which also helps explain the dynamic growth of the sac of IIA.^14^ The time interval between infectious emboli and IIA development has been estimated to be 24–48h.^15^ In our present case, we presented a DSA approved newly‐developed IIA that ruptured in 7 days. The case emphasized the importance of dynamic supervision on vascular conditions once the IE‐related IIA was diagnosed. CTA is now the initial choice for intracranial vascular image, as it is comparable to that of two‐dimensional DSA for detection of aneurysm.^5^ For special considering of pediatric population, including contrast administration and radiation exposure, magnetic resonance angiography (MRA) act as alternative choice comparable to CTA.

It seems difficult to predict the rupture of IIA, including the possibility and time. In considering the disaster consequence of aneurysm rupture, emergency surgery was recommended. The strategies in management of IIA has significantly changed throughout the last 3 decades from traditional open surgical approaches toward endovascular treatment, even in high‐volume, tertiary cerebrovascular referral centers.^6^, ^16^ It may be concluded that microsurgery has become a second‐line treatment option for IIAs in the pediatric population. Microsurgical trapping with involved vessel wall segment and pseudoaneurysm is the most common microsurgical technique for the treatment of a pediatric IIA. In case of IIA located in MCA bifurcation, end‐to‐end bypass technique used for adequate revascularization has been reported.^5^ In fact, endovascular therapy of IIA is utilized as the first option for pediatric patients, even at busy tertiary microvascular centers,^17^ through parent artery occlusion (PAO) with detachable coils, particles, or liquid embolic agents is the biggest concern. Interestingly, the use of PAO for children was reported actually to be safer than for adults. Gross et al. reported 100% occlusion rate for N‐butyl cyanoacrylate (NBCA) or Onyx embolization of IIA without any reported permanent complication or procedure‐related mortality in a meta‐analysis.^18^ In the present case, the first diagnosed IIA in DAR was successfully embolized by Onyx with magic 1.2 Fr. microcatheter without any complications obtained in the days following operation, even the microcatheter is not inside the sac of IIA but as close as possible. On the other hand, if the newly formed IIA in right bifurcation of MCA was detected ahead, the baby should be rescued from aneurysm rupture. In assumption, the newly formed IIA should burden a great risk of rebleeding in procedure because of the thin wall of pseudoaneurysm. PAO of the middle branch of the right MCA with coils should be the first choice. Without avoiding back stream of Onyx or NBCA, the neurological deficits because of limited infarction would recover like the left side. Even at the present situation, aneurysm clipping or raping could be done following evacuation of hematoma, but it is too risky and the whole branches of MCA should be occluded once rebleeding occurred during craniotomy. Evacuation of hematoma only with two‐stage operation of IIA is another choice.

## 4 CONCLUSION

IIAs are devastating complications with high mortality of pediatrics diagnosed with IE. The present case reported a newly formed and ruptured infectious intracranial aneurysm in few days following the first intracranial aneurysm embolization concurrent with MCA occlusion. Here, we suggest that the vascular condition should be dynamically supervised since one IIA was diagnosed. In addition, positive endovascular treatment of IIA in the early stage is recommended to avoid aneurysmal rupture and disaster in pediatrics.

## AUTHOR CONTRIBUTIONS

**Jun Tang**: Conceptualization; principal operator; writing‐reviewing and editing manuscript. **YunYing Yang**: Assistant; discussion and writing original draft. **Hongtu Ma and Hui Hu**: Assistant and image collection. **Lusheng Li:** Discussion; literature data analysis and Supervision.

## CONFLICT OF INTEREST STATEMENT

The authors declare that they have no conflict of interest.

## ETHICS STATEMENT

The study has been performed in accordance with the Declaration of Helsinki and approved by Human Subject Research Ethics Committee of Children's Hospital of Chongqing Medical University.

## CONSENT FOR PUBLICATION

Written informed consent for publication of their clinical details was obtained from the parent of the patient.

## Data Availability

The datasets analyzed during the current study are available from the corresponding author on reasonable request.
